# *Trypanosoma cruzi* and *Toxoplasma gondii* Induce a Differential MicroRNA Profile in Human Placental Explants

**DOI:** 10.3389/fimmu.2020.595250

**Published:** 2020-11-06

**Authors:** Lisvaneth Medina, Christian Castillo, Ana Liempi, Jesús Guerrero-Muñoz, Maura Rojas-Pirela, Juan Diego Maya, Humberto Prieto, Ulrike Kemmerling

**Affiliations:** ^1^ Programa de Anatomía y Biología del Desarrollo, Facultad de Medicina, Instituto de Ciencias Biomédicas, Universidad de Chile, Santiago, Chile; ^2^ Instituto de Biología, Pontificia Universidad Católica de Valparaíso, Valparaíso, Chile; ^3^ Programa de Farmacología Molecular y Clínica, Facultad de Medicina, Instituto de Ciencias Biomédicas, Universidad de Chile, Santiago, Chile; ^4^ Instituto de Investigaciones Agropecuarias, Ministerio de Agricultura, Santiago, Chile

**Keywords:** *Trypanosoma cruzi*, *Toxoplasma gondii*, human placental explants, miRNA profile, host gene expression****

## Abstract

*Trypanosoma cruzi* and *Toxoplasma gondii* are two parasites than can be transmitted from mother to child through the placenta. However, congenital transmission rates are low for *T. cruzi* and high for *T. gondii*. Infection success or failure depends on complex parasite-host interactions in which parasites can alter host gene expression by modulating non-coding RNAs such as miRNAs. As of yet, there are no reports on altered miRNA expression in placental tissue in response to either parasite. Therefore, we infected human placental explants *ex vivo* by cultivation with either *T. cruzi* or *T. gondii* for 2 h. We then analyzed the miRNA expression profiles of both types of infected tissue by miRNA sequencing and quantitative PCR, sequence-based miRNA target prediction, pathway functional enrichment, and upstream regulator analysis of differentially expressed genes targeted by differentially expressed miRNAs. Both parasites induced specific miRNA profiles. GO analysis revealed that the *in silico* predicted targets of the differentially expressed miRNAs regulated different cellular processes involved in development and immunity, and most of the identified KEGG pathways were related to chronic diseases and infection. Considering that the differentially expressed miRNAs identified here modulated crucial host cellular targets that participate in determining the success of infection, these miRNAs might explain the differing congenital transmission rates between the two parasites. Molecules of the different pathways that are regulated by miRNAs and modulated during infection, as well as the miRNAs themselves, may be potential targets for the therapeutic control of either congenital Chagas disease or toxoplasmosis.

## Introduction

More than one billion people worldwide are burdened by parasitic diseases (1). Of these, Chagas disease (American trypanosomiasis) and toxoplasmosis are caused by *Trypanosoma cruzi* (*T. cruzi*) and *Toxoplasma gondii* (*T. gondii*), respectively (2–4). Chagas disease is a devastating but neglected health problem in Latin America. Due to the extensive global migration of asymptomatic individuals, this infection has become an emerging disease in non-endemic countries. Congenital transmission is partially responsible for the progressive globalization of Chagas disease (5, 6). *T. gondii* is one of the most successful parasites on earth and is estimated to infect over one billion people worldwide (7). Importantly, both parasites can be congenitally transmitted and cause perinatal morbidity and mortality (2–4) but present different transmission rates. *T. cruzi* has a low transmission rate (1–12%) (6, 8) while *T. gondii* has a high transmission rate (22–72%) (3). Moreover, both parasites elicit a different local placental immune response that might be related to infection susceptibility (9, 10). Thus, *T. cruzi* and *T. gondi* infection is related to the expression and activation of different Toll-like receptors, which in turn mediate the secretion of different cytokines and chemokines in defense against both parasites in the placenta (9, 11).

However, the probability of congenital transmission depends on a variety of complex interactions between the pathogen and the host (4, 12). In particular, parasite factors, placental factors, and maternal and developing fetal immune systems determine infection occurrence (4, 13). In this context, both parasites display sophisticated strategies to avoid host defenses and virulence factors that increase the chance of establishing infection and long-term persistence. One of these strategies is the ability to modulate host cell gene expression (14–16) through small non-coding RNAs such as microRNAs (miRNAs) that repress mRNAs in a sequence-specific manner by either an mRNA degradation process or through mRNA translation inhibition (17–19). MiRNAs play a key role in fine tuning gene expression in multiple physiological and pathological conditions including *T. cruzi* (20) or *T. gondii* (21) infection. Interestingly, the largest miRNA cluster in humans is encoded in chromosome 19 (C19MC; 19q13.41) and is almost exclusively expressed in the placenta (22). Both C19MC-derived and non-C19MC-derived miRNAs have been associated with placental development pathologies such as pre-eclampsia and infection (23, 24). However, there is no report in the literature regarding altered miRNA expression in placental tissue in response to either parasite.

Here, we infected human placental explants (HPE) *ex vivo* by 2 h of incubation with either *T. cruzi* or *T. gondii*, then analyzed both miRNA expression profiles by miRNA sequencing and quantitative PCR of selected miRNAs. In addition, we used sequence-based miRNA target prediction and performed pathway functional enrichment and upstream regulator analysis of differentially expressed genes targeted by differentially expressed miRNAs (DEMs).

## Materials and Methods

### Parasite Culture and Harvesting

For *T. cruzi*, Y strain (*T. cruzi* II) trypomastigotes were obtained from previously infected Vero cells (ATCC^®^ CCL-81) grown in RPMI medium supplemented with 5% fetal bovine serum and 1% antibiotics (penicillin-streptomycin) at 37°C in a humid atmosphere with 5% CO_2._ Parasites invaded the cells and replicated intracellularly as amastigotes. After 48–72 h, amastigotes transformed back to trypomastigotes and lysed the host cells. The infective trypomastigotes were separated from cellular debris by low speed centrifugation (500 × *g*) for 10 min. Parasites were isolated from the supernatant by centrifugation at 3500×*g* during 15 min, suspended in RPMI media (without fetal bovine serum, 1% (penicillin-streptomycin) (RPMI 1640^®^, Biological Industries Ltd.), and quantified in a Neubauer chamber (9).

For *T. gondii*, semi-confluent HFF cells were infected with RH tachyzoites at a multiplicity of infection of 3 to 5 parasites per cell. After 40 h, the infected cells were washed, then monolayers were scraped from the flasks and passed through 20-, 23-, and 25-gauge needles. Tachyzoites were purified from host cell debris with a 3.0 μm Isopore filter (Merck Millipore^®^) (25).

The laboratory has been certificated as a Biosafety level 2 laboratory by the Biosafety Committee (“Unidad de Prevención de Riesgo”) of the “Facultad de Medicina, Universidad de Chile” (approval # 0403/2019).

### HPE Infection

Human term placentas were obtained from 3 women with uncomplicated pregnancies with vaginal or caesarean delivery. Informed consent for experimental use of the placenta was given by each patient as stipulated by the Code of Ethics of the “Servicio de Salud Metropolitana Norte” (approval number 0010/2019). Exclusion criteria consisted of the following: major fetal abnormalities, placental tumor, intrauterine infection, obstetric pathology, positive serology for Chagas disease, and any other maternal disease. Donor patients were negative for anti-*T. gondii* IgG/IgM antibodies. The organs were collected in cold, sterile, saline-buffered solution (PBS) and processed no more than 30 min after delivery. The dissected explants (approximately 50 mg of tissue) were washed with sterile PBS to remove the blood and co-cultivated with *T. cruzi* trypomastigotes or *T. gondii* tachyzoites (10^5^ parasites/ml) in serum free RPMI media. After 2 h of co-cultivation, explants were collected in RNAlater™ solution (ThermoFisher Scientific^®^), then stored at 4°C for 24 h and at -80°C for posterior RNA isolation (9). Three independent experiments were carried out in triplicates; HPEs from each placenta were infected with either *T. cruzi* or *T. gondii* parasites. The parasite load in the HPEs was confirmed by real-time PCR as described previously by us (9, 10, 26).

### RNA Extraction

Total RNA was extracted from HPE by mechanical disruption in 1.3 ml of RNA-solv^®^ reagent (Omega Bio-tek) and isolated using an E.Z.N.A^®^ total RNA kit I (Omega Bio-tek) according to manufacturer instructions. RNA was stored at -80°C until analysis. The concentration and quality of RNA was determined with a Qubit^®^ RNA HS Assay kit and an IQ Assay kit (Invitrogen), respectively. Only RNA samples with an IQ ≥8 were further analyzed for quality with an Agilent 2100 Bioanalyzer System (Agilent Technologies, USA) using an RNA Nano 6000 Assay Kit. RNA samples with RNA integrity numbers >5.0 were used for miRNA profiling analysis (9).

### Library Construction and Sequencing

Small RNA-Seq libraries were constructed with an Illumina TruSeq Small RNA library preparation kit according to manufacturer protocols. To assess the quality of the libraries, a DNA High Sensitivity Chip was used in an Agilent 2100 Bioanalyzer (Agilent Technologies, USA). The libraries were sequenced on an Illumina NextSeq 500 platform. For each condition, three independent biological replicates were sequenced and paired-end reads were generated.

### Data Analysis

Raw read quality was evaluated using the FastQC tool (https://www.bioinformatics.babraham.ac.uk/projects/fastqc/). Raw reads were analyzed with Trim Galore Cutadapt software (27) and low-quality reads were removed (phred value < 30) in order to obtain clean reads. Clean reads with a length range of 18–35 nucleotides were chosen to perform all subsequent analyses. The sofware STAR (28) was used to align all reads to the reference human genome sequence (Hg38). The read counts per coding sequence were determined using HTSeq-count (29). To evaluate replicates, we used Principal Component Analysis, Pearson correlation and standardized median correlation analyses and box plots. The program EdgeR was used for differential expression analysis (30). Differentially expressed genes were defined as genes with p-value <0.05. Target gene prediction performed by using miRDB, psRNA target, and TargetScan sofwares.

### Enrichment Analyses

miRNA set enrichment analysis was performed using the TAM 2.0 tool (http://www.lirmed.com/tam2/). KEGG pathways and functional annotation of the predicted target genes (https://www.genome.jp/kegg/kegg2.html) were also analyzed to determine the biological processes, molecular functions, cellular components, and associations with disease.

### RT-qPCR

RNA enriched in small RNAs was extracted from HPEs (approximately 50 mg of tissue) by mechanical disruption in 1 ml RNAzol^®^ RT (Sigma-Aldrich) according to manufacturer instructions and stored at -80°C until analysis. The concentration of the isolated miRNAs was determined using a Qubit^®^ Quant-iT™ microRNA Assay Kit (Molecular Probes). cDNA of mature miRNAs was synthesized with a MystiCq™ microRNA cDNA Synthesis Mix Kit (Sigma-Aldrich Merck) per manufacturer guidelines. The 25 µl RT-qPCR reaction contained 12.5 µl 2× MystiCq microRNA SYBR Green qPCR Ready Mix, 0.5 µl of 10 µM MystiCq Universal PCR Primer, 0.5 µl of 10 µM of each specific MystiCq microRNA qPCR Assay Primer (**Supplementary Table 1**), 10.5 µl nuclease-free water, and 1 µl cDNA. All RT-qPCR reactions were performed in triplicates. RT-qPCR was performed under the following cycling conditions: initial denaturation at 95°C for 2 min, followed by 40 cycles of 95°C for 5 s and 60°C for 30 s. Gene expressions were calculated using the 2^−ΔΔCT^ relative expression method and normalized to snRNA U6 (RNU6-1) expression levels (31).

## Results

### *T. cruzi* and *T. gondii* Change the miRNA Expression Profile in HPE

**Figure 1:**
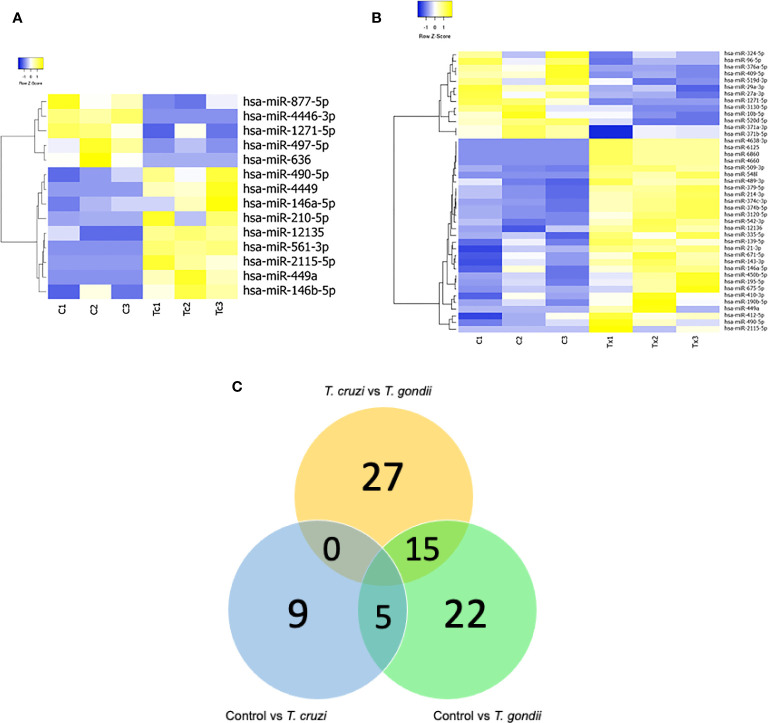
Expression profiling of mature miRNAs in human placental explants (HPEs) following *T. cruzi* or *T. gondii* infection. **(A)** Heat-map of differentially expressed microRNAs (DEMs) in control vs. *T. cruzi* infected HPE and **(B)** control vs. *T. gondii* infected HPE. The filtered miRNA data were subjected to unsupervised hierarchical clustering analysis. The metric was set as the Euclidean distance. Control: C1, C2, C3; *T. cruzi:* Tc1, Tc2, Tc3; *T. gondii:* Tx1, Tx2, Tx3. **(C)** Venn diagram showing the number of commonly expressed and specifically expressed miRNAs between the infected HPE groups. Significant miRNAs for HPE with *T cruzi* infection vs. those with *T. gondii* infection are shown in the yellow circle. The blue circle represents the miRNAs that discriminate between the control (uninfected HPE) and *T. cruzi* infected HPE, while the green circle represents the miRNAs that distinguish the control (uninfected HPE) and *T. gondii* infected HPE.

The effects of *T. cruzi* and *T. gondii* on placental tissue were assayed in HPE after a 2 h challenge with 10^5^ parasites/ml. Total miRNA extracted from infected and non-infected control HPE was analyzed by miRNA Seq. Key characteristics of the obtained sequencing data are summarized in **Table 1**. A total of 680 and 686 DEMs were identified in *T. cruzi* and *T. gondii* infected HPE, respectively. Only 14 DEMs with a minimum 1.5-fold change in expression and a 95% probability of being differentially expressed (p ≤ 0.05) were identified in *T. cruzi* challenged samples (**Figure 1A**). In *T. gondii* challenged samples, the number of DEMs increased to 42 (**Figure 1B**). Comparison of *T. cruzi* infected HPE with non-infected control samples showed that five miRNAs were downregulated and nine were upregulated. In *T. gondii* infected tissues, 13 miRNAs were downregulated and 29 were upregulated. The Venn diagram in **Figure 1C** shows the miRNAs that were differentially expressed in the presence of both parasites compared to non-infected control samples and in HPE infected with either parasite. The complete list of DEMs in response to *ex vivo* *T. cruzi* and *T. gondii* infection is shown in **Table 2**.

**Table 1:** Statistics of the small RNA sequences obtained in this study.

	M reads (millions)	M Aligned (millions)	% aligned	Mature microRNA reads	# Mature MicroRNAs	# Mature MicroRNA (single aligned)
**S1 Control**	12,96	11,68	90,12	3489287	922	732
**S2 Control**	15,17	13,87	91,43	4855933	938	760
**S3 Control**	15,58	13,16	84,50	3030989	843	714
**S4 *T. cruzi***	14,35	12,60	87,77	3000672	907	712
**S5 *T. cruzi***	14,76	12,94	87,69	3910944	907	737
**S6 *T. cruzi***	16,13	13,15	81,55	3571933	914	720
**S7 *T. gondii***	13,57	11,82	87,07	3160630	916	737
**S8 *T. gondii***	14,40	12,79	88,77	4747358	962	757
**S9 *T. gondii***	13,51	12,16	89,99	4148647	931	763

M reads, total of sequences in analysis after raw data processing (millions); M aligned, total of mapped sequences to the human genome (millions); % aligned, total of mapped sequences to the human genome (percentage); Mature microRNA reads, mapped reads counted as mature miRNAs; # Mature microRNAs, number of mature miRNAs; # Mature MicroRNA (single aligned), number of mature miRNAs associated to unique miRNA precursors in the reference.

**Table 2:** The top differentially expressed miRNAs (P < 0.05) in HPE challenged with 10^5^ *T. cruzi* trypomastigotes or *T. gondii* tachyzoites during 2 h.

	miRNAs	Fold Change	p-value	p-adjustment	Expression
Control vs *T. cruzi*	hsa-miR-490-5p	1,47644601	0,011939305	1	Up-regulated
	hsa-miR-497-5p	-0,95822045	0,021380502	1	Down-regulated
	hsa-miR-146a-5p	0,9111643	0,024398238	1	Up-regulated
	hsa-miR-12135	2,76826832	0,025125553	1	Up-regulated
	hsa-miR-210-5p	1,17471038	0,038576256	1	Up-regulated
	hsa-miR-146b-5p	0,70985057	0,041583753	1	Up-regulated
	hsa-miR-877-5p	-0,71572824	0,049301458	1	Down-regulated
	hsa-miR-1271-5p	-1,48497011	0,049553349	1	Down-regulated
*T. cruzi* (*treatment exclusive)	hsa-miR-636		0,003652701	1	Up-regulated
	hsa-miR-4449		0,005197851	1	Up-regulated
	hsa-miR-449a		0,009529598	1	Up-regulated
	hsa-miR-2115-5p		0,013101594	1	Up-regulated
	hsa-miR-561-3p		0,023021063	1	Up-regulated
	hsa-miR-4446-3p		0,041796581	1	Up-regulated
Control vs *T. gondii*	hsa-miR-12136	1,79576197	0,00017626	0,120736654	Up-regulated
	hsa-miR-335-5p	1,35817451	0,0007489	0,227732906	Up-regulated
	hsa-miR-10b-5p	-1,51399053	0,00099737	0,227732906	Down-regulated
	hsa-miR-1271-5p	-2,41452099	0,00206894	0,267855922	Down-regulated
	hsa-miR-409-5p	-2,51430487	0,00225422	0,267855922	Down-regulated
	hsa-miR-27a-3p	-0,97356696	0,00234618	0,267855922	Down-regulated
	hsa-miR-29a-3p	-0,92877383	0,00344997	0,337603687	Down-regulated
	hsa-miR-214-3p	0,9642214	0,00435186	0,372627686	Up-regulated
	hsa-miR-379-5p	1,46136088	0,00619518	0,453658878	Up-regulated
	hsa-miR-3120-5p	1,05085403	0,00662276	0,453658878	Up-regulated
	hsa-miR-376a-5p	-1,06317073	0,00928715	0,578335925	Down-regulated
	hsa-miR-542-3p	1,36466429	0,01456701	0,760274481	Up-regulated
	hsa-miR-195-5p	1,29637989	0,01520981	0,760274481	Up-regulated
	hsa-miR-3130-5p	-2,64645685	0,01847345	0,760274481	Down-regulated
	hsa-miR-519d-3p	-0,91993812	0,0188483	0,760274481	Down-regulated
	hsa-miR-490-5p	1,42743639	0,01886813	0,760274481	Up-regulated
	hsa-miR-450b-5p	1,11411811	0,02263153	0,763303449	Up-regulated
	hsa-miR-374b-5p	0,86524386	0,02512358	0,763303449	Up-regulated
	hsa-miR-374c-3p	0,87017312	0,02523843	0,763303449	Up-regulated
	hsa-miR-143-3p	0,78495077	0,02900217	0,763303449	Up-regulated
	hsa-miR-21-3p	0,79317052	0,0291757	0,763303449	Up-regulated
	hsa-miR-675-5p	1,1546043	0,03213608	0,763303449	Up-regulated
	hsa-miR-671-5p	1,76630085	0,03293839	0,763303449	Up-regulated
	hsa-miR-146a-5p	0,86261284	0,03359957	0,763303449	Up-regulated
	hsa-miR-489-3p	1,02467619	0,03372793	0,763303449	Up-regulated
	hsa-miR-96-5p	-2,45982286	0,03878464	0,77095716	Down-regulated
	hsa-miR-509-3p	0,86545472	0,03909277	0,77095716	Up-regulated
	hsa-miR-190b-5p	2,26556495	0,04749263	0,77095716	Up-regulated
	hsa-miR-371b-5p	-0,92943309	0,04875339	0,77095716	Down-regulated
	hsa-miR-520d-5p	-0,6667811	0,04979001	0,77095716	Down-regulated
	hsa-miR-371a-3p	-0,92563764	0,04993063	0,77095716	Down-regulated
	hsa-miR-412-5p	0,86533956	0,0514002	0,77095716	Up-regulated
	hsa-miR-324-5p	-1,01010797	0,05178532	0,77095716	Down-regulated
	hsa-miR-410-3p	0,63475365	0,05378966	0,77095716	Up-regulated
	hsa-miR-139-5p	0,66901369	0,0543406	0,77095716	Up-regulated
*T. gondii* (*treatment exclusive)	hsa-miR-548l		0,01756271	0,760274481	Up-regulated
	hsa-miR-449a		0,02617767	0,763303449	Up-regulated
	hsa-miR-6125		0,03454366	0,763303449	Up-regulated
	hsa-miR-4638-3p		0,03454366	0,763303449	Up-regulated
	hsa-miR-6860		0,03454366	0,763303449	Up-regulated
	hsa-miR-4660		0,03454366	0,763303449	Up-regulated
	hsa-miR-2115-5p		0,04354449	0,77095716	Up-regulated

*Treatment Exclusive: refers to miRNAs sequenced only in the T. cruzi or T. gondii infected condition (treatment), they are not expressed in control explants.

### Functional Annotation and KEGG Pathway Enrichment Analysis of miRNA Target Genes

**Figure 2:**
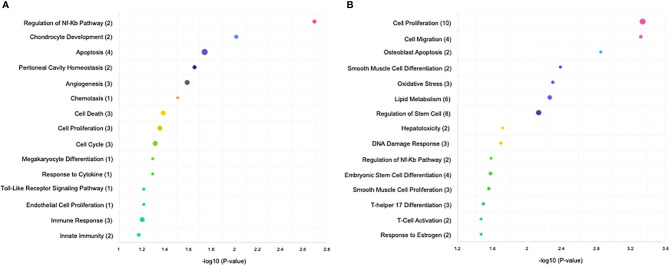
KEGG analysis of the differentially expressed miRNAs (P < 0.05) revealed significant enrichment in immune-related and cell cycle pathways. The top 15 enriched pathways of the differentially expressed miRNAs are presented for **(A)** control vs. *T. cruzi* and **(B)** control vs. *T. gondii*. The sizes and colors of the circles represent the number of predicted gene targets of the differentially expressed miRNAs (P < 0.05) and the q-value, respectively.

To better understand the roles of the miRNAs identified in HPE in response to *ex vivo* infection with both parasites, the target genes of the miRNAs were identified using miRDB, psRNA target, and TargetScan. GO and KEGG enrichment analyses used to identify the biological functions of the DEMs (p < 0.05) during *ex vivo* *T. cruzi* and *T. gondii* infection revealed 679 best scored target genes of the 14 miRNAs from *T. cruzi* vs. control, 1970 best scored target genes of the 42 miRNAs from *T. gondii* vs. control, and 2011 best scored target genes of the 42 miRNAs from *T. cruzi* vs. *T. gondi*. The target genes of the DEMs are shown in **Supplementary Table 2**. Among the significantly enriched GO terms in *T. cruzi* vs. control samples, DEMs were significantly enriched in regulation of NFκB pathways, chondrocyte development, cell death including apoptosis, peritoneal cavity homeostasis, angiogenesis, cell cycle, megakaryocyte differentiation, Toll-like receptor signaling pathway, and immune response including innate immunity (**Figure 2A**). Among the significantly enriched GO terms in *T. gondii* vs. control samples, DEMs were significantly enriched in cell proliferation, cell migration, osteoblast differentiation, oxidative stress, lipid metabolism, regulation of stem cells including embryonic stem cells, hepatotoxicity, DNA damage response, regulation of NFκB pathways, smooth muscle proliferation, T-helper 17 cell differentiation, T-cell activation, and response to estrogen (**Figure 2B**).

**Figure 3:**
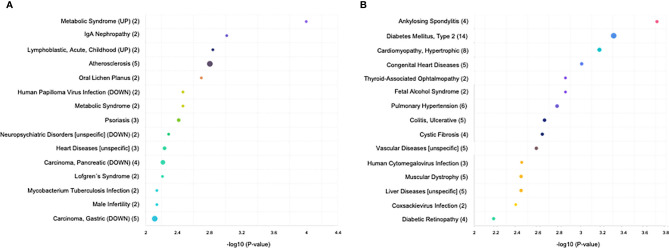
KEGG analysis of the differentially expressed miRNAs (P < 0.05) revealed significant enrichment in cancer-related, infections, and inflammatory disease pathways. The top 15 enriched pathways of the differentially expressed miRNAs are presented for **(A)** control vs. *T. cruzi* and **(B)** control vs. *T. gondii*. The sizes and colors of the circles represent the number of predicted gene targets of the differentially expressed miRNAs (P < 0.05) and the q-value, respectively.

In addition, we performed GO and KEGG analyses to identify different pathologies in which the *T. cruzi*- and *T. gondii*-induced DEMs were related. In *T. cruzi* vs. control samples, significantly enriched DEMs were related to metabolic syndrome, IgA-nephropathy, acute childhood lymphoblastic leukemia, atherosclerosis, oral lichen planus, human papilloma virus infection, psoriasis, neuropsychiatric disorders, heart diseases, pancreatic carcinoma, Löfgren’s syndrome, *Mycobacterium tuberculosis* infection, male infertility, and gastric carcinoma (**Figure 3A**). In *T. gondii* vs. control samples, the significantly enriched DEMs were related to ankylosing spondylitis, type 2 diabetes mellitus, hypertrophic cardiomyopathy, congenital heart disease, fetal alcohol syndrome, pulmonary hypertension, ulcerative colitis, cystic fibrosis, vascular diseases, human cytomegalovirus infection, muscular dystrophy, liver diseases, coxsackievirus infection, and diabetic retinopathy (**Figure 3B**).

**Figure 4:**
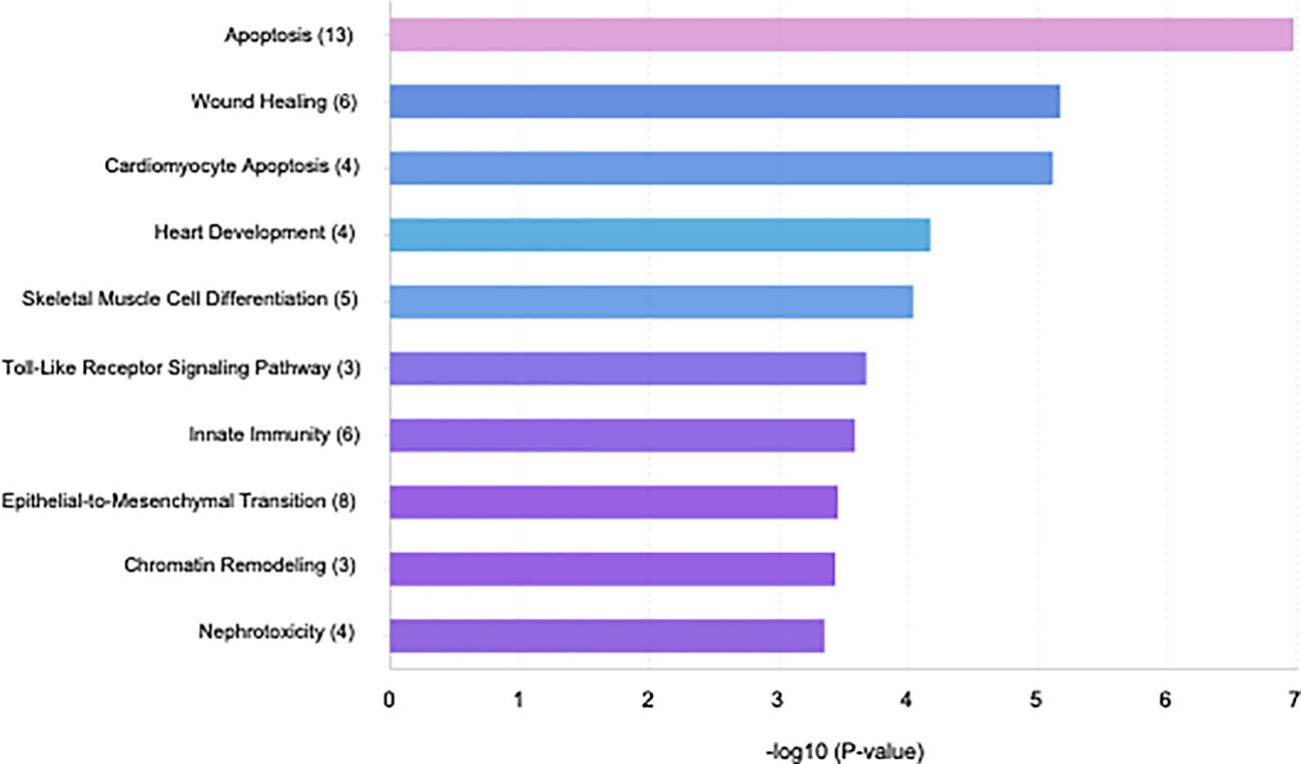
Bar plot illustrating the top 10 significant miRNA-function associations of the sequenced miRNA related to placental and/or parasitic diseases in human placental explants infected with *T. cruzi* or *T. gondii*.

**Figure 5:**
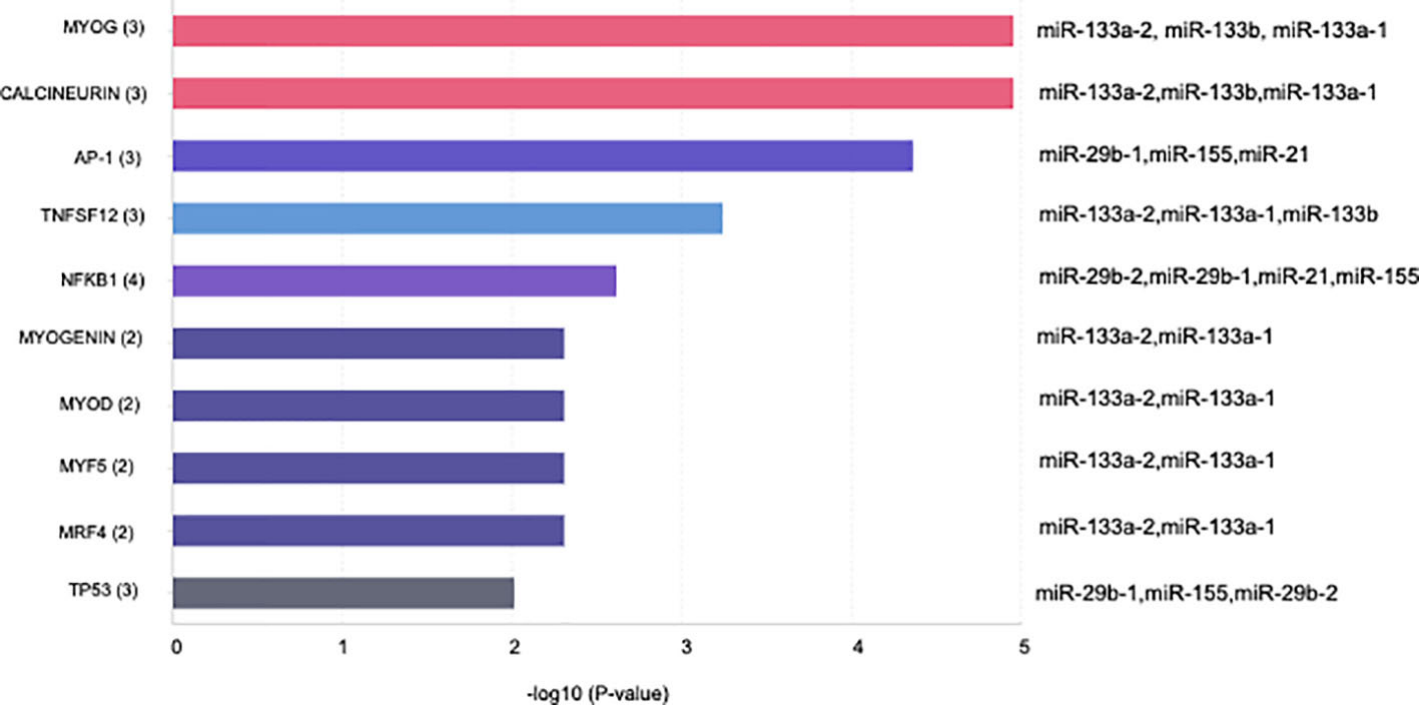
Bar plot illustrating the top 10 significant miRNA transcription factor associations of the sequenced miRNA related to placental and/or parasitic diseases in human placental explants infected with *T. cruzi* or *T. gondii*.

Specific GO and KEGG enrichment analyses focused on the functions of DEMs in response to both parasites that were related to parasitic diseases and/or placenta pathology. These DEMs are listed in **Table 3**. In this context, we found that the significantly enriched DEMs were related to the regulation of apoptosis, wound healing, cardiomyocyte apoptosis, heart development, skeletal muscle cell differentiation, Toll-like receptor signaling pathway, innate immunity, epithelial to mesenchymal transition, chromatin remodeling, and nephrotoxicity (**Figure 4**). Moreover, we analyzed the significantly enriched DEMs related to transcription activation and found that the following transcription factors or its activators were modulated by them: MYOG, calcineurin, AP-1, TNFSF12, NFκB1, myogenin, MYOD, MYF5, MRF4, and TP53 (**Figure 5**).

**Table 3:** Sequenced miRNA related to placental and/or parasitic diseases in HPE challenged with 10^5^ *T. cruzi* trypomastigotes or *T. gondii* tachyzoites during 2 h.

miRNAs	Control vs T. cruzi		Control vs T. gondii	
	Expression	p-value	Expression	p-value
hsa-miR-3074	Up-regulated	0,282778175	Down-regulated	0,906716427
hsa-miR-518e-5p	Down-regulated	0,736493282	Up-regulated	0,655921033
hsa-miR-127-3p	Down-regulated	0,454473692	Up-regulated	0,899001399
hsa-miR-512-3p	Up-regulated	0,893095693	Up-regulated	0,554888541
hsa-miR-516a-5p	Down-regulated	0,573579895	Down-regulated	0,875664943
hsa-miR-376a-3p	Up-regulated	0,26239456	Up-regulated	0,890873441
hsa-miR-523-5p	Down-regulated	0,736493282	Up-regulated	0,655921033
hsa-miR-517-5p	Down-regulated	0,356215385	Down-regulated	0,131367096
hsa-miR-523-3p	Up-regulated	0,558965508	Down-regulated	0,070624754
hsa-miR-519a-5p	Down-regulated	0,902087668	Up-regulated	0,683786458
hsa-miR-526a-5p	Down-regulated	0,878621126	Up-regulated	0,975623088
hsa-miR-519a-3p	Up-regulated	0,855200309	Down-regulated	0,216046946
hsa-miR-518e-3p	Up-regulated	0,878596629	Down-regulated	0,062749763
hsa-miR-520c-5p	Down-regulated	0,878525243	Up-regulated	0,981710662
hsa-miR-526a-3p	Down-regulated	0,648457618	Down-regulated	0,194744348
hsa-miR-29b-3p	Up-regulated	0,688264908	Down-regulated	0,461394462
hsa-miR-520c-3p	Down-regulated	0,492741665	Down-regulated	0,321148465
hsa-miR-133a-3p	Up-regulated	0,800691009	Down-regulated	0,140142195
hsa-miR-525-5p	Down-regulated	0,892625222	Down-regulated	0,624806979
hsa-miR-525-3p	Up-regulated	0,896175408	Down-regulated	0,075998808
hsa-miR-519c-5p	Down-regulated	0,736493282	Up-regulated	0,655921033
hsa-miR-518b	Up-regulated	0,991656949	Down-regulated	0,078353573
hsa-miR-519c-3p	Down-regulated	0,262008953	Down-regulated	0,786169155
hsa-miR-520e-5p	Down-regulated	0,485165729	Up-regulated	0,996288443
hsa-miR-520e-3p	Up-regulated	1	Up-regulated	0,818473073
hsa-miR-21-5p	Up-regulated	0,783933507	Up-regulated	0,129614377
hsa-miR-21-3p	Up-regulated	0,749764521	Up-regulated	0,029175701
hsa-miR-517a-3p	Up-regulated	0,596271375	Down-regulated	0,461347018
hsa-miR-519e-5p	Up-regulated	0,105129034	Up-regulated	0,757374294
hsa-miR-519e-3p	Up-regulated	0,385269175	Up-regulated	0,69099671
hsa-miR-518d-5p	Down-regulated	0,878525243	Up-regulated	0,981710662
hsa-miR-520g-5p	Up-regulated	0,892300156	Up-regulated	0,751979117
hsa-miR-518d-3p	Up-regulated	0,55414416	Down-regulated	0,942071295
hsa-miR-520b-5p	Down-regulated	0,421187791	Down-regulated	0,477328076
hsa-miR-520g-3p	Up-regulated	0,728189158	Down-regulated	0,352658846
hsa-miR-519a-2-5p	Down-regulated	0,421187791	Down-regulated	0,477328076
hsa-miR-520b-3p	Up-regulated	0,530509712	Down-regulated	0,954450249
hsa-miR-517c-3p	Up-regulated	0,760586655	Down-regulated	0,675509383
hsa-miR-524-5p	Down-regulated	0,490615316	Down-regulated	0,115381834
hsa-miR-210-5p	Up-regulated	0,038576256	Up-regulated	0,576691951
hsa-miR-204-5p	Up-regulated	0,62227226	Down-regulated	0,583793777
hsa-miR-524-3p	Down-regulated	0,907890609	Down-regulated	0,099098809
hsa-miR-519b-5p	Down-regulated	0,736493282	Up-regulated	0,655921033
hsa-miR-210-3p	Up-regulated	0,497217414	Down-regulated	0,843820092
hsa-miR-378a-5p	Down-regulated	0,915728534	Down-regulated	0,504825517
hsa-miR-526b-5p	Down-regulated	0,501400731	Down-regulated	0,534886926
hsa-miR-519b-3p	Up-regulated	0,996536077	Up-regulated	0,986254335
hsa-miR-518a-5p	Down-regulated	0,396658878	Down-regulated	0,099042516
hsa-miR-520d-5p	Down-regulated	0,163923317	Down-regulated	0,049790011
hsa-miR-526b-3p	Up-regulated	0,690372369	Down-regulated	0,356016985
hsa-miR-520d-3p	Down-regulated	0,368956264	Down-regulated	0,595399092
hsa-miR-30e-3p	Up-regulated	0,708643074	Down-regulated	0,606142557
hsa-miR-520h	Up-regulated	0,420508027	Down-regulated	0,48103803
hsa-miR-519d-5p	Down-regulated	0,699132481	Down-regulated	0,210250996
hsa-miR-515-5p	Down-regulated	0,451420159	Down-regulated	0,479490879
hsa-miR-519d-3p	Up-regulated	0,795099209	Down-regulated	0,018848296
hsa-miR-515-3p	Down-regulated	0,961837732	Down-regulated	0,056222245
hsa-miR-518c-5p	Down-regulated	0,433629597	Down-regulated	0,461615584
hsa-miR-155-5p	Up-regulated	0,089556012	Up-regulated	0,307144182
hsa-miR-518c-3p	Up-regulated	0,825417746	Down-regulated	0,393062915
hsa-miR-520a-5p	Up-regulated	0,829237058	Down-regulated	0,786085152
hsa-miR-376a-5p	Down-regulated	0,101845272	Down-regulated	0,009287146
hsa-miR-520a-3p	Up-regulated	0,303331111	Down-regulated	0,733502488
hsa-miR-144-5p	Up-regulated	0,979441392	Down-regulated	0,781688613
hsa-miR-204-5p	Up-regulated	0,62227226	Down-regulated	0,583793777
hsa-miR-424-5p	Up-regulated	0,52797867	Down-regulated	0,318765339
hsa-miR-346	Down-regulated	0,996223653	Up-regulated	0,484560011

### Validation of miRNA Expression by RT-qPCR

**Figure 6:**
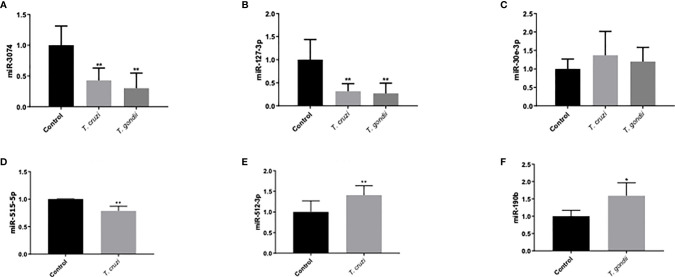
Validation of miRNAs related to placental and/or parasitic diseases using RT-qPCR. Human placental explants were challenged with 10^5^ *T. cruzi* trypomastigotes or *T. gondii* tachyzoites for 2 h. The presence of miRNA was determined by real-time PCR. *T. cruzi* and *T. gondii* decreased miR-3074 and miR-127-3p expression, while no change was observed in miR-30e-3p expression **(A–C)**. *T. cruzi* inhibited miR-515-5p expression and induced miR512-3p **(D, E)**. *T. gondii* induced miR-190b-5p **(F)**. All values are the mean ± S.D. and correspond to at least three independent experiments that were performed in triplicate. Data were normalized in terms of the control values and analyzed by Student’s t-test or ANOVA. *p ≤ 0.05; **p ≤ 0.01.

Validation of miRNAs associated with parasite infection or pregnancy related pathologies was performed by selecting six miRNAs [miR-3074 (26), miR-127-3p (27, 28), miR-30e-3p (29), miR-512-3p (30), miR-515-5p (31), and miR-190b (32)] for confirmation by real time PCR to verify the DEM expression levels. Expression of miR-3074, miR-127-3p, and miR-30e-3p (**Figures 6A–C**) was analyzed in HPE in response to both parasites. miR-512-3p and miR-515-5p (**Figures 6D–E**) expression was determined in response to *T. cruzi* infection and miR-190b expression in response to *T. gondii* infection (**Figure 6F**). All selected miRNAs except for miR-30e-3p (**Figure 6C**) were differentially expressed. Thus, miR-3074 expression (**Figure 6A**) was significantly decreased (*T. cruzi*: 57.03 ± 19.99%, p ≤ 0.01; *T. gondii*: 69.84 ± 24.67%, p ≤ 0.01) with respect to the control but not the infected samples. Decreased miR-3074 expression was expected in the *T. gondii* infected samples but not in the *T. cruzi* infected samples. According to the miRNA Seq data, miR-3074 was upregulated in *T. cruzi* challenged samples. Similar results were observed for miR-127-3p (**Figigure 6B**). Expression of miR-127-3p was significantly decreased in HPE infected with either parasite (*T. cruzi*: 68.218 ± 16.41%, p ≤ 0.01; *T. gondii*: 73.13 ± 22.45%, p ≤ 0.01) compared to the control but not to infected samples; we expected an increase in miR-127-3p expression in the presence of *T. gondii* since in the miRNA Seq data this particular miRNA was increased (**Table 3**). RT-qPCR validation results for miR-512-3p, miR-515-5p, and miR-190b confirmed the miRNA Seq data. Thus, miR-512-3p expression increased (40.83 ± 22.53%, p ≤ 0.01) (**Figure 6D**) and miR-515-5p expression decreased (21.44 ± 8.60%, p ≤ 0.01) (**Figure 6E**) significantly in *T. cruzi* infected samples. In *T. gondii* infected HPE, miR-190b expression was significantly increased (59.02 ± 37.73%, p ≤ 0.01) (**Figure 6F**).

## Discussion

Pathogens have evolved strategies to exploit resources from their hosts to maximize their own survival, replication, and dissemination. Thus, different kinds of pathogens (including parasites) have developed sophisticated mechanisms that include hijacking host cellular machinery to modulate host gene expression to inhibit defense responses (16, 32, 33). Both of the parasites studied here are able to manipulate host gene expression. For instance, during *T. cruzi* cell and tissue invasion, cell reprogramming affects cellular stress responses, host metabolism, and a significant number of transcription factors (16, 34). *T. gondii* also reprograms host cells, primarily targeting cell-specific transcription factors that regulate host defenses (i.e., NF-κB, interferon regulatory factor, and JAK/STAT) by regulating their intrinsic activities and expression levels (35). In addition, certain parasites including *T. cruzi* and *T. gondii* can alter host miRNA expression to favor both parasite clearance and infection (18, 19). Moreover, different strains of *T. gondii* can induce specific miRNAs in mice that have been proposed as biomarkers for early infection (19, 36).

Mature miRNAs regulate the expression of over 30% of fundamental genes; these are involved in key biological processes including development, cellular proliferation and differentiation, apoptosis, metabolism, and immune response (18, 19, 37); all of these determine infection success or failure. Moreover, epigenetic and genetic defects in miRNAs and their processing machinery are a common hallmark of infection and diseases that include pregnancy-specific pathologies such as preeclampsia (17, 38).

Most of the transcriptomic studies as well as those analyzing miRNA profiles have focused on a single type of cell response (34) or on tissues or organs in animal models (39, 40); no studies have focused on human tissues. The present study is the first report on the miRNA profile of the human placenta in response to *T. cruzi* or *T. gondii* infection. We identified 680 and 686 DEMs, respectively, in *T. cruzi* and *T. gondii* infected samples. *T. cruzi* modulated only 14 DEMs with a minimum of a 1.5-fold change in expression and a 95% probability of being differentially expressed (**Figure 1A**). In contrast, the number of DEMs increased to 42 in *T. gondii* challenged HPE (**Figure 2B**). Our results showed that the DEMs identified here are related to the regulation of different fundamental cellular processes (**Figure 2**) as well as different pathologies (**Figure 3**). Moreover, fundamental cellular processes related to placenta pathologies and embryonic development are affected by the presence of both parasites (**Figures 4**–**5**). It is important to point out, that tissue response to infection is relevant during disease progression. The presence of the parasites leads to tissue damage as well as immune and regulatory/repair responses, which can lead to fibrosis and tissue dysfunction as observed in chagasic cardiomyopathy (41) or *Toxoplasma* induced encephalitis in immune-compromised individuals (42).

Three miRNAs, miR-21, miR-146a/b, and miR-210, were overrepresented in most of the ontology terms (**Table 2**, **Supplementary Tables 3**–**4**). Previous studies have implicated these miRNAs in immune and inflammatory response regulation *via* macrophage polarization controlled through transcription factor regulation in response to signals from the microenvironment (43, 44). Concordantly, in *T. cruzi*-infected mice, increased miR-21 expression in the heart has been correlated with a parasitemia peak at 30 days post-infection (39). In placenta, miR-21 has been associated with trophoblast differentiation and invasion and miR-21 dysregulation leads to placental pathology (45). MiR-146a is a negative feedback regulator in TLR-4 signaling that acts by repressing TRAF6 to inhibit NFκB transcription factor activation (46, 47). In macrophages, TRAF6 mediates the induction of the pro-inflammatory cytokine IL-12, which is essential to control *T. gondii* infection (48). TRAF6 activation is also required for vacuole-lysosome fusion, a fundamental step during *T. gondii* infection (49). Our results showed that in HPEs, *T. gondii* and *T. cruzi* infection increased miR-146a expression. Our previous studies showed that both parasites modulate placental immune response differentially through TLRs and NFκB pathways in HPEs (9, 10) Interestingly, the inhibition of these pathways increased the DNA loads of both parasites in HPEs (10). Increased *T. gondii* infection in placental tissue is also induced by TLR-4 inhibition (9). In addition, increased levels of miR-146a have been reported in the brains of mice infected with *T. gondii*, moreover, miR-146a ablation affects early parasite burden and improves survival (50). It was previously reported that miR-210 is induced by damage associated molecular patterns (51). In preeclamptic placentas, miR-210 is increased (52); in the present study, miR-210 was increased in HPE infected with *T. cruzi* but not with *T. gondii*. Expression of miR-210 can be directly regulated by the specific binding of NF-κB p50 to its putative promoter (53). In this context, it is important to mention that *T. cruzi*, but not *T. gondii*, infection of HPE activates both NF-κB signaling pathways (10). Therefore, the increased level of miR-210 might be a placental response to signal transduction pathway activation.

In addition, several identified pathways, important, e.g. for chondrocyte development, megakaryocyte smooth and muscle cell differentiation, hepatotoxicity, and DNA damage response, are neither related to infection or with placental tissues (**Figure 3**). This can be explained be the fact, that miRNAs target multiple genes, while individual genes are targeted by multiple miRNAs. Moreover, the same miRNA regulates different genes in different tissues and organs (54, 55). Here, we chose to validate six miRNAs that were associated specifically with parasite infection and/or pregnancy related pathologies (**Figure 6**). Deregulation of miR-30e-3p has been reported in mice that were experimentally infected with *T. gondii* (40). This miRNA is also related to Chagas cardiomyopathy (39) and is upregulated in placentas with intrauterine growth restriction (56). Nonetheless, miR-30e-3p expression was unaffected by *T. cruzi* or *T. gondii* infection in HPE (**Figure 6C**). Increased miR-3074-5p expression has been described in placentas from recurrent miscarriages (57) and in livers from *T. gondii-*infected cats (42). However, miR-3074-5p expression was diminished in HPE infected either with *T. cruzi* or *T. gondii* (**Figure 6A**). The differences between our results and the reported data might be explained by differences in the studied organs (heart and liver *versus* placenta) and the complexity of the above mentioned placental pathologies. MiR-127 is a placenta-specific miRNA codified in the C14MC cluster (58) and its levels are decreased in placenta-related pathologies such as recurrent miscarriage and small-for-gestational age (59, 60); the downregulation of MiR-127 was also detected in babies infected congenitally with either parasite (3, 4). Concordantly, our results showed that HPE infection with either *T. cruzi* or *T. gondii* led to the decrease of this miRNA (**Figure 6B**). Moreover, a decreased expression of miR-127-3p in non-placental tissues has been reported during *T. gondii* infection in mice and cats (40, 42, 61), but there is no report regarding miR-127-3p expression in response to *T. cruzi* infection. Both miR-515-5p and miR-512-3p are placenta-specific miRNAs that are codified in the C19MC cluster (62). Decreased miR-515-5p expression is related to fetal growth restriction (63) and preeclampsia (64). Importantly, this miRNA inhibits human trophoblast differentiation by directly repressing the aromatase P450 (*CYP19A1*), frizzled 5 (*FZD5*), and glial cells missing 1 transcription factor (*GCM1*) genes (65). Trophoblast differentiation is part of the trophoblast epithelial turnover and it has been proposed that this mechanism is part of an antiparasitic placental response against *T. cruzi* infection (11, 13, 66, 67). Therefore, our reported decrease of miR-515-5p expression during *ex vivo* *T. cruzi* infection of HPE (**Figure 6D**) might be at least partially responsible for the parasite-induced trophoblast differentiation. In contrast, miR-512-3p was upregulated in HPE in response to *T. cruzi* infection (**Figure 6E**). Interestingly, miR-512-3p confers resistance to vesicular stomatitis virus in non-placental recipient cells (68) and represses the caspase 8 inhibitor c-FLIP (cellular FLICE-like inhibitory protein); it consequently increases caspase 8 activity (69). Caspase 8 regulates trophoblast differentiation and apoptotic cell death and is activated by *T. cruzi* (66). Therefore, miR-512-3p upregulation might also be a protective placental response to *T. cruzi* infection, as it is to viral infection. The upregulation of miR-190b in HPE during *T. gondii* infection was observed in the RNAseq analysis (**Figure 1B**, **Table 3**), then validated by qPCR (**Figure 6F**). Upregulation of miR-190b promotes cell proliferation and migration and reduces cell apoptosis in different types of cancer (70, 71). Parasites modulate apoptotic responses in infected cells to avoid rapid clearance; *T. gondii* is particularly capable of blocking apoptosis by different mechanisms (33). In neurons, increased miR-190b expression also increases cell viability, suppresses autophagy, and significantly decreases the levels of pro-inflammatory TNF-α, IL-6, and IL-1β cytokines (72). In this context, we have shown that *T. gondii*, in contrast to *T. cruzi*, does not induce pro-inflammatory cytokines in HPE (9). Therefore, it is postulated that the lack of pro-inflammatory cytokine secretion in response to *T. gondii* in HPE might be related to an increase in miR-190b expression and that, together with the modulation of the apoptotic pathway, it could allow parasite persistence and infection in the placenta.

**Figure 7:**
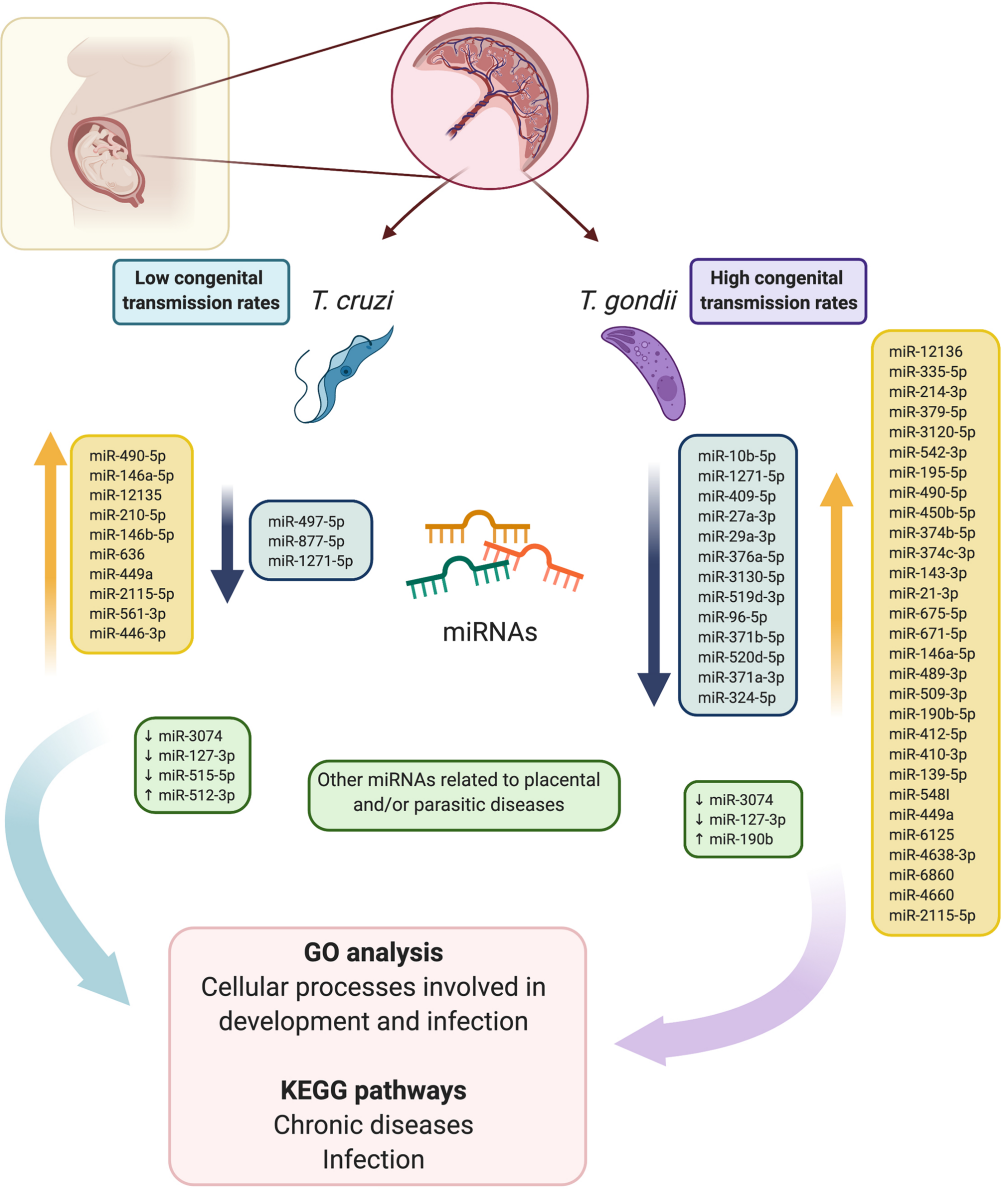
Schematic summary of the comparative analysis of RNA sequencing-based miRNA profiles in HPEs in response to *ex vivo* *T. cruzi* and *T. gondii* infection. Fourteen, and 42 DEMs were identified in *T. cruzi* and *T. gondii* infected samples. In *T. cruzi* infected HPEs, 5 miRNAs were downregulated and 9 were upregulated. In *T. gondii* infected HPEs, 13 miRNAs were downregulated and 29 were upregulated. In addition, five miRNAs that are associated specifically with parasite infection and/or pregnancy-related pathologies were validated. GO analysis revealed that the predicted targets of the DEMs were different cellular processes involved in development and immunity, and most of the identified KEGG pathways were related to chronic diseases and infection. Considering that the DEMs identified herein modulate crucial host cellular targets potentially determining the success of infection, these miRNAs might explain the differences in the congenital transmission rates of the two parasites. This figure was created using BioRender.com.

In conclusion, the present study provides a comparative analysis of RNA sequencing-based miRNA profiles in HPE in response to *ex vivo* *T. cruzi* and *T. gondii* infection (**Figure 7**). Our findings provide new insight into the capacity of both parasites to modulate host gene expression. GO analysis revealed that the predicted targets of the DEMs were different cellular processes involved in development and immunity, and most of the identified KEGG pathways were related to chronic diseases and infection. Considering that the DEMs identified herein modulate crucial host cellular targets that participate in determining the success of infection, these miRNAs might explain the differences in congenital transmission rates. Molecules of the different pathways that are regulated by miRNAs and modulated during infection, as well as the miRNAs themselves, may be potential targets for the therapeutic control of either congenital Chagas disease or toxoplasmosis.

## Data Availability Statement

The RNA-seq data reported in the present study have been submitted to the NCBI SRA database (http://www.ncbi.nlm.nih.gov/bioproject/656620; accession number PRJNA656620). All other data supporting the findings can be found in the article/**Supplementary Material**.

## Ethics Statement

The studies involving human participants were reviewed and approved by Ethical Committee of the “Servicio de Salud Metropolitano Norte” Santiago de Chile, Chile. The patients/participants provided their written informed consent to participate in this study.

## Author Contributions

LM and UK conceived of and planned experiments. LM, CC, MR-P, AL, and JG-M carried out experiments. JM and HP contributed to the interpretation of the results. UK and LM wrote the manuscript. All authors contributed to the article and approved the submitted version.

## Funding

UK received a grant from the Network of the European Union, Latin America and the Caribbean Countries on Joint Innovation and Research Activities (ERANet-LAC; grant number ERANet17/HLH-0142). UK, JM, and CC received grants from the National Fund for Scientific and Technological Development (FONDECYT; grant numbers 1190341, 1170126, and 3180452, respectively).

## Conflict of Interest

The authors declare that the research was conducted in the absence of any commercial or financial relationships that could be construed as a potential conflict of interest.
