# Supplementary material for: Trypanosoma cruzi and Toxoplasma gondii Induce a Differential MicroRNA Profile in Human Placental Explants
Source: Front Immunol. 2020 Nov 6;11:595250. doi: 10.3389/fimmu.2020.595250 (PMC7677230; doi:10.3389/fimmu.2020.595250)
Supplement: Supplementary file 1 [file Table_1.docx]

Supplementary Table 1. Oligonucleotides used as primers for miRNA-specific qRT-PCR analysis.

| **qPCR Assay Primers** | **Sequence** |
| --- | --- |
| miR-127-3p | UCGGAUCCGUCUGAGCUUGGCU |
| miR-30e-3p | CUUUCAGUCGGAUGUUUACAGC |
| miR-3074 | GAUAUCAGCUCAGUAGGCACCG |
| miR-512-3p | AAGUGCUGUCAUAGCUGAGGUC |
| miR-515-5p | UUCUCCAAAAGAAAGCACUUUCUG |
| miR-190b | UGAUAUGUUUGAUAUUGGGUU |
| RNU6-1 | GUGCUCGCUUCGGCAGCACAUAUACUAAAAUUGGAACGAUACAGAGAAGAUUAGCAUGGCCCCUGCGCAAGGAUGACACGCAAAUUCGUGAAGCGUUCCAUAUUUU |
